# Efficient IoT-Based Control for a Smart Subsurface Irrigation System to Enhance Irrigation Management of Date Palm

**DOI:** 10.3390/s21123942

**Published:** 2021-06-08

**Authors:** Maged Mohammed, Khaled Riad, Nashi Alqahtani

**Affiliations:** 1 Date Palm Research Center of Excellence, King Faisal University, Al-Ahsa 31982, Saudi Arabia; nalqahtani@kfu.edu.sa; 2 Agricultural Engineering Department, Faculty of Agriculture, Menoufia University, Shebin El Koum 32514, Egypt; 3 Department of Computer Science, College of Computer Sciences and Information Technology, King Faisal University, Al-Ahsa 31982, Saudi Arabia; 4 Department of Mathematics, Faculty of Science, Zagazig University, Zagazig 44519, Egypt; khaled.riad@science.zu.edu.eg; 5 Department of Food and Nutrition Sciences, College of Agricultural and Food Sciences, King Faisal University, P.O. Box 420, Al-Ahsa 31982, Saudi Arabia

**Keywords:** Internet of Things, microcontroller, water productivity, sensor-based, subsurface irrigation scheduling, evapotranspiration, remote monitoring, micro-irrigation control

## Abstract

Drought is the most severe problem for agricultural production, and the intensity of this problem is increasing in most cultivated areas around the world. Hence improving water productivity is the primary purpose of sustainable agriculture. This study aimed to use cloud IoT solutions to control a modern subsurface irrigation system for improving irrigation management of date palms in arid regions. To achieve this goal, we designed, constructed, and validated the performance of a fully automated controlled subsurface irrigation system (CSIS) to monitor and control the irrigation water amount remotely. The CSIS is based on an autonomous sensors network to instantly collect the climatic parameters and volumetric soil water content in the study area. Therefore, we employed the ThingSpeak cloud platform to host sensor readings, perform algorithmic analysis, instant visualize the live data, create event-based alerts to the user, and send instructions to the IoT devices. The validation of the CSIS proved that automatically irrigating date palm trees controlled by the sensor-based irrigation scheduling (S-BIS) is more efficient than the time-based irrigation scheduling (T-BIS). The S-BIS provided the date palm with the optimum irrigation water amount at the opportune time directly in the functional root zone. Generally, the S-BIS and T-BIS of CSIS reduced the applied irrigation water amount by 64.1% and 61.2%, respectively, compared with traditional surface irrigation (TSI). The total annual amount of applied irrigation water for CSIS with S-BIS method, CSIS with T-BIS method, and TSI was 21.04, 22.76, and 58.71 m^3^ palm^−1^, respectively. The water productivity at the CSIS with S-BIS (1.783 kg m^−3^) and T-BIS (1.44 kg m^−3^) methods was significantly higher compared to the TSI (0.531 kg m^−3^). The CSIS with the S-BIS method kept the volumetric water content in the functional root zone next to the field capacity compared to the T-BIS method. The deigned CSIS with the S-BIS method characterized by the positive impact on the irrigation water management and enhancement on fruit yield of the date palm is quite proper for date palm irrigation in the arid regions.

## 1. Introduction

Water scarcity in arid and semi-arid regions is a primary constraint to sustainable cultivation [1]. However, ongoing population growth requires sustained growth of food production in the future, which in turn requires irrigation water inputs that can support irrigated cultivation. Even though the utilization of reclaimed water and desalinated seawater can facilitate agricultural irrigation, the safety risk of reclaimed water, the high-energy requirement, and the high cost of desalination water hinder this irrigation utilization [2,3,4]. Therefore, there is a need to use innovative irrigation technologies to ensure sufficient food production with optimum irrigation water amount [1,5,6].

Date palm (*Phoenix dactylifera* L.) is dominant in the arid and semi-arid regions characterized by water resource scarcity [7,8,9]. Despite water scarcity in these regions, inefficient irrigation water use still prevails on the date palm orchards, which is indicated for the precious groundwater sources depletion [10]. The authors in [11] estimated the irrigation water requirements of date palm trees in different areas of Saudi Arabia. They mentioned that the amount of irrigation water required for the date palm per hectare (100 palms ha^−1^) ranged from 7299 to 9495 m^3^ ha^−1^, based on the proportion of the date palm cultivated area. Another study was carried in the western region of Saudi Arabia; they determined the amount of irrigation water of 7300 m^3^ ha^−1^ for the date palm (100 palms ha^−1^) [12]. There is an urgent worldwide need to change from traditional irrigation systems to on-demand irrigation methods for water conservation in these regions [13]. Although the highest yield of the date palm is obtained when providing complete irrigation water requirements by traditional surface irrigation methods, the exact yield can be achieved with significantly more limited irrigation water application by using modern irrigation systems [14]. Therefore, continuous efforts to design modern irrigation systems improve irrigation water management, crop yield, and reducing irrigation water inputs [1,6,15,16,17].

Most currently applied irrigation systems are based on constant periodical water application regardless of the plant’s actual need for irrigation water, which is a big challenge [5]. Proper irrigation scheduling assists in producing good crop production. However, when surface irrigation systems are applied, it is not practical to change the irrigation water depth and frequency too much due to the difficulty of implementation. Variations in irrigation depth are very confusing for the producers to vary the irrigation schedule [18,19]. Determining the optimum amount of water is not an easy task; it depends on multiple factors such as air temperature, an average of relative humidity, wind speed, sun hours, and solar radiation [19,20]. Therefore, the irrigation depth is roughly estimated to keep the irrigation depth and the interval constant over the growing period [18]. The accurate determination of the irrigation schedule is a complicated and time-consuming process. Using computer programs has made it more accessible, and it is now possible to schedule the irrigation water amount precisely according to the water required for the crops [14,18,21]. Sensor-based irrigation scheduling is an efficient tool customized to field characteristics that can significantly facilitate irrigation scheduling decisions [4].

Due to the enormous revolution of IoT and the development of sensors for intelligent agriculture, its applications introduce a significant positive impact on plants and irrigation water conservation [22]. The authors in [23] presented an IoT-based monitoring and control system for irrigation. They focused on the facilitation of supplying adequate water amount to some domestic crops in India. The authors used a pumping mechanism to apply the water needed by the soil. However, they did not use a flow meter to measure the amount of water applied. Also, in [23], the authors considered the surface irrigation system that wastes enormous water because of water evaporation.

Moreover, the deep roots of the date palm have not benefited from all these wasted amounts of water in surface irrigation. In our CSIS, we automated the on-demand irrigation by using a subsurface irrigation system. Thus, we reached the optimum amount of water required by the date palm tree. Moreover, we eliminated the evaporation caused by surface irrigation. The authors in [21] introduced an IoT-based dynamic irrigation scheduling system used for efficient water management. The authors focused on automating the irrigation process instead of the manual irrigation treatment. They measured the water level that real-time presents in the field with a low-cost sensor. The introduced system collects the measures and introduces them to the farmer to decide on the manual irrigation method. There should exist some recommendations/suggestions because such a vast model must not depend on the farmers experience.

Improved monitoring for the measures affecting the irrigation system for mustard leaf is introduced in [24]. The authors employed an IoT monitoring system using ESPresso Lite V2.0 module that connected with a VH400 sensor, a flow meter, and a weather station. The introduced framework in [16] is for monitoring only; no actions are taken based on the monitored data. Another study [25] controlled the volume and frequency of irrigation water based on an irrigation water management system and some low-cost sensors for measuring the soil moisture level. The authors in [25] used a FC-28 soil moisture sensor. Machine learning is also used with IoT for automating farming. The authors in [26] introduced automation for the irrigation system used by farmers to increase the yield and quality. The authors constructed a wireless sensor network in each region on the farm. Machine learning is used to predict irrigation patterns depending on the crops and some weather scenarios. On the other hand, the authors have clarified neither the employed machine learning algorithms nor the technical structure of the wireless sensor network.

Precision agriculture has also employed deep learning neural networks with IoT for intelligent irrigation systems [6]. It is considered a feedback system that works better based on the weather of any region. In this system, the water deficit is observed by farmers experience based on the leaf color of the plant and the growth of branches. This must be done using machine vision to avoid errors caused by human observations.

Subsurface irrigation systems are considered the most effective way to conserve irrigation water. The irrigation water does not evaporate from the soil surface using subsurface irrigation systems compared to traditional surface irrigation methods. The subsurface irrigation systems are characterized by water application at low intensity directly in the functional root zone of the date palm [1,16,27,28,29]. The automatic irrigation system, including wireless soil moisture sensors, obtains more yield with less irrigation water requirement, and the water productivity is improved [5,17,25]. The efficient irrigation systems considerably help increase the water-productivity of crops, especially in regions with water deficits and a lack of automated irrigation facilities [30,31].

Due to the importance of water and its scarcity in arid regions, it is imperative to employ modern technologies and intelligent systems for improving water productivity and conservation. Although subsurface irrigation systems can save irrigation water, these systems must be developed to avoiding the disadvantages of traditional surface and subsurface irrigation methods. In the traditional subsurface irrigation systems, it is necessary to have one of the workers constantly in the field for follow-up the irrigation processes, this is very cumbersome for farmers in practice. Moreover, the applied water amount is usually imprecision due to those the subsurface irrigation systems are largely unseen. There is also a need to overcome the possibility that the wetting pattern may be too small in the soil due to irrigation system breakdown or insufficient irrigation water necessary. Thus, we should emphasize using smart subsurface irrigation systems. Moreover, employing the promising capabilities for the Internet of Things (IoT) and cloud platforms to control subsurface irrigation systems. To improve water productivity and conservation, avoid the problems of the traditional subsurface irrigation systems, and enhance the yield of date palm in the arid region. This is behind our motivation to design, construct, and validate the fully automated subsurface irrigation system to control the water amount needed for irrigating date palm trees in the arid regions.

The main goal of this study is to implement a fully automated subsurface irrigation system with sensor-based/time-based irrigation scheduling methods for irrigation water conservation in arid regions. To achieve this goal, we divided it into four sub-objectives:

- Design and construct a modern subsurface irrigation system for date palm irrigation.
- Design and install an autonomous sensors’ network to instantly collect the volumetric moisture content for sensor-based irrigation scheduling and climatic parameters for time-based irrigation scheduling.
- Employ the ThingSpeak cloud platform to host our data, perform algorithmic analysis, create event-based alerts to the user, create event-based alerts to the user, and send instructions to the IoT devices of the applied irrigation scheduling method.
- Study the impact of the controlled subsurface irrigation system on irrigation water consumption, water productivity, and yield of date palm compared with applied traditional surface irrigation system in the experimental area.

## 2. Materials and Methods

### 2.1. Experimental Site

The controlled subsurface irrigation system (CSIS) was designed and constructed at the Date Palm Research Center of Excellence (DPRC, King Faisal University, Al-Ahsa, Saudi Arabia). The experiment was conducted in an arid region at the DPRC farms (Latitude: 25.261° N, Longitude: 49.708° E) for one year from 1 January to 31 December 2020. The electrical conductivity (EC_w_), pH, and total dissolved solids (TDS) of the used irrigation water in the experiment were 0.93 ± 0.11 dS m^−1^, 7.8 ± 0.8, and 685 ± 58.5 mg L^−1^. Table 1 shows the physical and hydraulic properties of the sandy loam soil in the experimental site.

### 2.2. Description of the Controlled Subsurface Irrigation System

**Figure 1:**
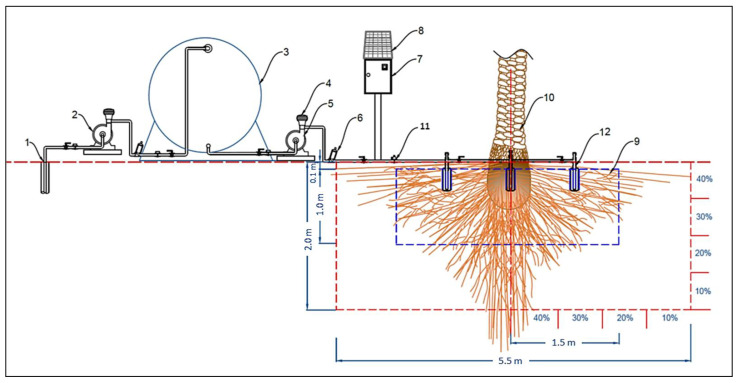
Schematic diagram of the essential components of the designed subsurface irrigation system (SIS). (1) Groundwater well, (2) Water pump, (3) Water tank, (4) Automatic water pump regulator, (5) Water pump, (6) Disc filters, (7) Control unit, (8) Solar panel, (9) Target root zone, (10) Date palm trunk, (11) Solenoid valve, (12) Subsurface irrigation unit.

The controlled Subsurface Irrigation System (CSIS) consisted of the subsurface irrigation system with its mechanical and electrical parts and the remote monitoring and controlling system with its electronic hardware and software components. Figure 1 shows the main components of the CSIS, and below is the description of these main components of the CSIS:

#### 2.2.1. Design of Subsurface Irrigation System

**Figure 2:**
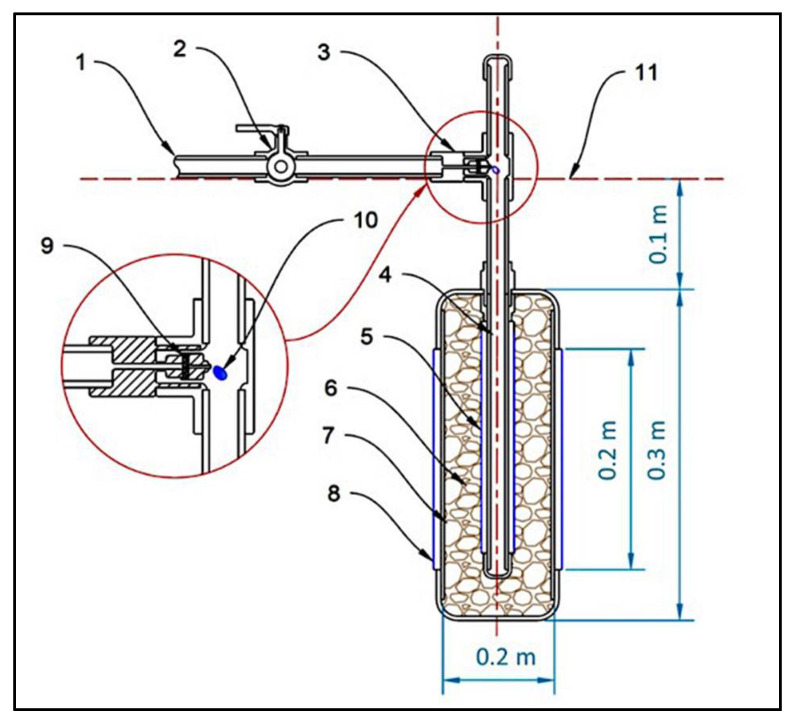
Schematic diagram of the designed subsurface irrigation unit used in the controlled subsurface irrigation system (dimensions in m). (1) Water source, (2) Manual valve, (3) Water flow regulator, (4) Perforated inner pipe, (5) Filtering cloth of the inner pipe, (6) light volcanic gravel, (7) Slotted outer pipe, (8) Filtering cloth of the outer pipe, (9) Twistable dripper head, (10) Water droplet, (11) Ground level.

The subsurface irrigation system SIS was composed of a water resource, water pump set, irrigation network, power source, and control unit subsurface irrigation units (SIU). Details of the essential components of the CSIS are as follows:

- Water source and pump set: The source of water used for the experiment was from a groundwater well at the site of the experiment. The water is pumped from the well to the water tank using a 2 kW water pump. The water tank was made of polyethylene and had a volume of 5 m^3^. Another water pump (1 kW) was used to supply the designed irrigation network with sufficient irrigation water at the required pressure.
- Irrigation network: The irrigation network included the mainline, sub mains, and feeder ring pipe made of high-density polyethylene (HDPE) with diameters of 0.05 m, 0.03 m, and 0.025 m. The irrigation network also the disc filters (120 mesh, 130 microns), manual valves, solenoid valves, among other irrigation accessories.
- Power source and control unit: The power source of the control unit that included the electronic devices and power source of sensors were taken from a battery (12 V, 55 Ah). A 50 W solar panel charges this battery with a charging regulator.
- Subsurface irrigation unit (SIU): The SIU consisted of two perforated pipes made from polyvinyl chloride (PVC) wrapped with a filtering cloth to prevent the transfer of fine soil inside the unit, as shown in Figure 2.

**Figure 3:**
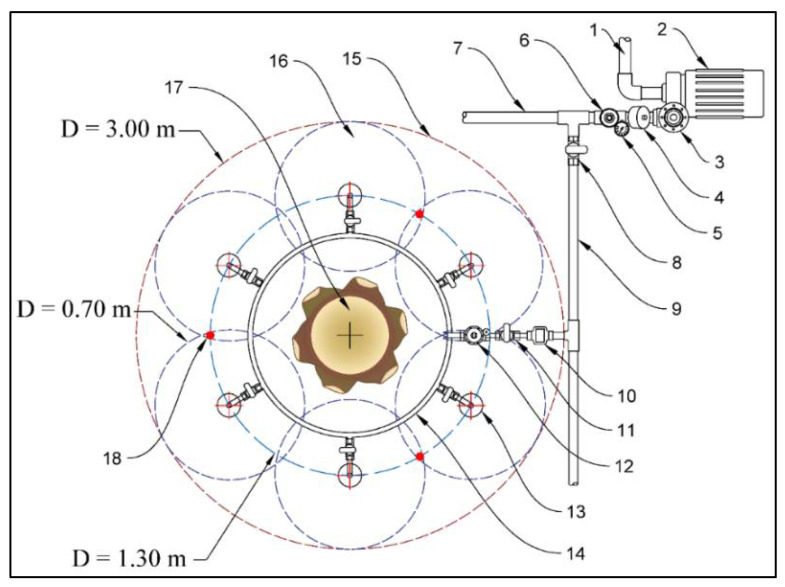
Typical layout of the six subsurface irrigation units distributed around the date palm tree. (1) Water inlet pipe (from the water tank), (2) Water pump, (3) Automatic water pump regulator, (4) Disc filter, (5) Pressure gauge, (6) Pressure regulator, (7) Irrigation mainline, (8) Manual valve, (9) Irrigation sub mainline, (10) Water flow meter, (11) Manual valve, (12) Solenoid water valve, (13) Subsurface irrigation unit, (14) Ring distribution pipe, (15) The irrigation target area, (16) The irrigation target area of each subsurface irrigation unit, (17) Date palm tree, (18) Position of the volumetric soil moisture sensor.

The length of the outer pipe was 0.30 m with a diameter of 0.10 m and slotted with a tilt angle of 30°. The slot width and length were 0.002 m and 0.004 m. The length of the inner pipe was 0.33 mm with a diameter of 0.025 m. The inner pipe was perforated in a spiral shape with a hole diameter of 0.03 m. Light volcanic gravel with a size of 0.004–0.008 m was placed between the two pipes to reduce the amount of water inside the unit, allowing all the nutrients to seep through. The flow rate of the SIU was adjusted using a low-pressure adjustable irrigation dripper (0–0.070 m^3^ h^−1^) at 0.030 m^3^ h^−1^ by twisting the dripper head at the irrigation network pressure of 250 k Pa. Six SIU were buried around each date palm tree at a circle of 1.30 m, as shown in Figure 3. The six SIUs were attached with the ring distribution pipe; the ring pipe was connected to the sub mainline above ground.

#### 2.2.2. IoT System Architecture

**Figure 4:**
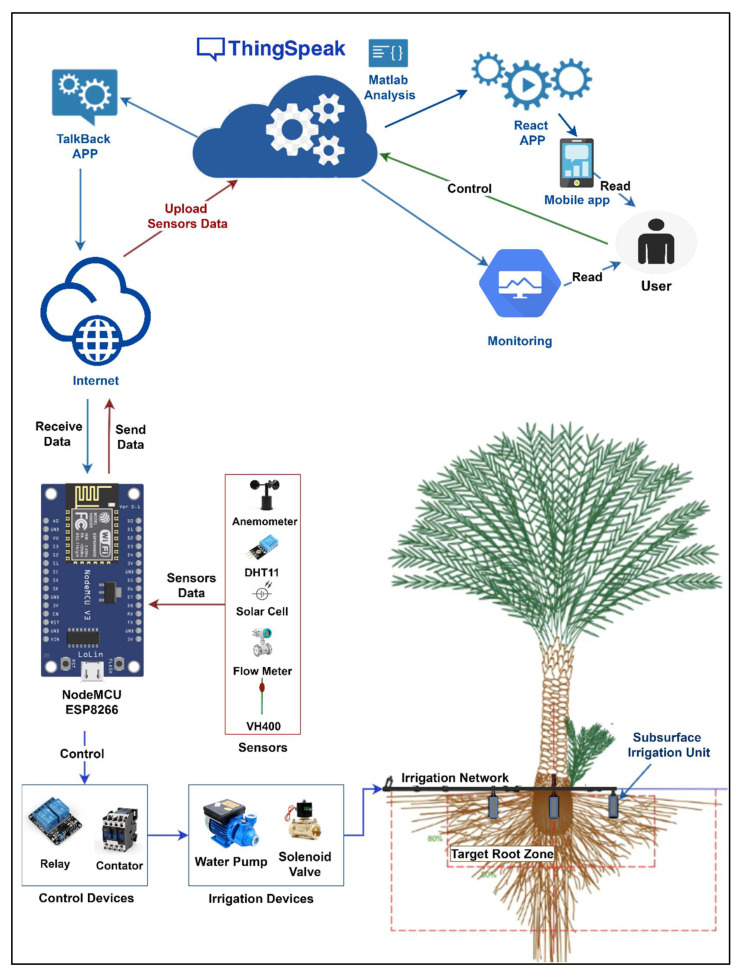
The designed controlled subsurface irrigation system (CSIS) was deployed around a date palm tree in our experimental field.

The main goal of the designed CSIS cloud IoT solution is water management of date palm by efficiently controlling the subsurface irrigation system through employing the marvelous capabilities of cloud computing and the IoT. Implicitly, the designed CSIS can monitor various parameters and automatically notify the user in case of emergency by either sending an email message or a short message. It controls the subsurface irrigation system for date palm to apply the optimum amount of water needed by the date palm tree. It can be considered as a sensor-based subsurface irrigation scheduling (S-BIS). It schedules the water amount to be applied for the date palm on variable periods based on the measures received from the sensors. The system gets the measurements from the sensors, uploads these measures to the ThingSpeak cloud platform, does cloud analysis, makes decisions, and applies decisions to the subsurface irrigation system. Our CSIS is shown in Figure 4. The designed system makes irrigation decisions based on direct measurement of volumetric water content (VWC), while monitoring other factors such as air temperature, relative humidity (RH), solar radiation, wind speed, and water flow rate per minute.

The figure shows the detailed workflow for our designed CSIS. It has five main components:

- Experimental field: we conducted our experiments (for the CSIS cloud IoT solution) over nine-date palm trees divided into three replications (R1, R2, and R3). The sensors of the designed system are deployed around the center palm tree for each replicate.
- Electrical and electronic devices: we employed the following electrical and electronic devices for each replicate in our experimental field—the NodeMCU (ESP8266 Shenzhen Quine Trading Co., Ltd., Shenzhen, China) as a microcontroller unit. We used a water pump, a solenoid valve, an anemometer, and a flow meter.
- Internet in the study area: to provide the Internet in the study area, we used a data SIM card of a local communications network and a 4G Router (HUAWEI, Hunan JENET Technology Co., Ltd. Changsha, China. As soon as the SIM card is plugged in HUAWEI 4G router and turn it on, the NodeMCUs instantly have Wi-Fi access. Then the NodeMCUs immediately connect to the Internet. The router was plugged all-time with a 5 V power source using an inverter (12 V to 5 V, 2.1 A) connected with the battery of our system. No problems with internet connection were observed in the study area during the trial period. The used router can connect up to 32 wireless devices, providing a fast and stable connection for all used NodeMCUs.
- A set of sensors: we employed three VH400 sensors (Vegetronix, Inc., Riverton, Salt Lake County, UT, USA) for each replicate. It is used to measure the VWC. Each of these VH400 sensors is installed between two subsurface irrigation (SIS) units at 0.8 m from the date palm tree trunk and 0.3 m depth. We used two DHT11 sensors for measuring relative humidity percentage and air temperature in the study area. Also, we measured the solar radiation by installing two solar cells under the shadow of the center date palm tree.
- ThingSpeak cloud platform: we upload the measurements collected from the set of sensors by using the ESP8266 module to our private channel on the ThingSpeak cloud platform. We employed *MATLAB Analysis, TalkBack App,* and *React App* (Mathematical computing software, Natick, MA, USA) to make decisions through the cloud platform.
- Monitoring interface: the user uses this to monitor the graphical data generated by our private channel on the ThingSpeak cloud platform.

Our CSIS is initialized by connecting the ESP8266 module to the Internet, setting up the smart connected sensors, and launching our private channel on the ThingSpeak cloud platform. Each corresponding sensor sends its real-time measures to the ESP8266 module, which uploads the collected measurements to our private channel on the ThingSpeak cloud platform.

The efficiently controlled water management process is achieved in our designed CSIS by a comprehensive analysis of the uploaded measurements on our private channel. At first, the MATLAB Analysis App runs an introduced algorithm to calculate new data based on the current data in the fields of our private channel. Based on the analysis above, some instructions, commands, or messages will be sent back to the ESP8266 module or the user using Talkback App or React App, respectively. Then, the ESP8266 module directly forwards the command/s to the designated connected sensor or device to execute the given instructions:

(1) Hardware layout

**Figure 5:**
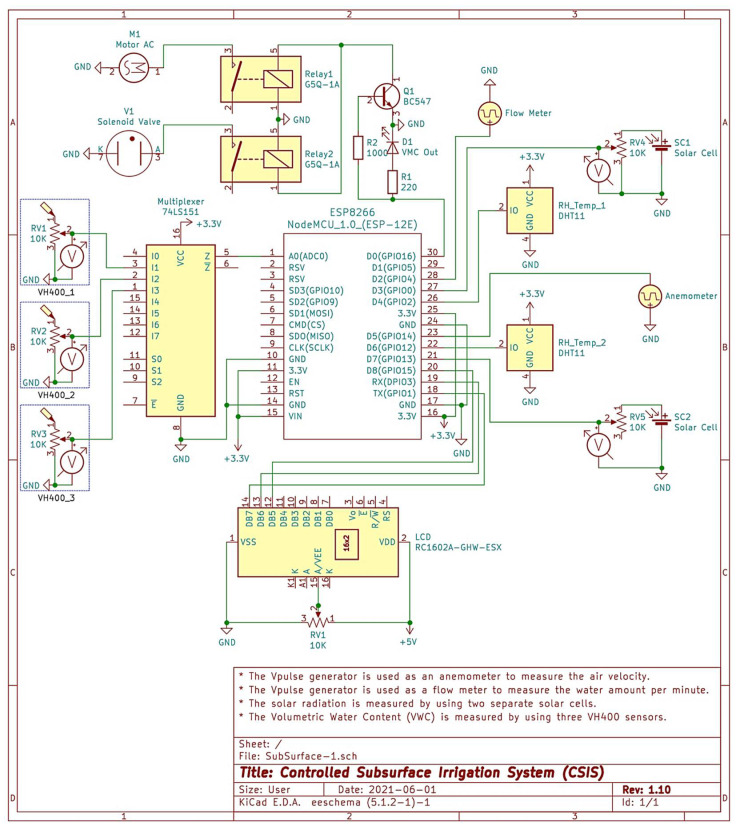
The detailed schematic KiCad diagram with successful electrical rules check.

The design phase for the expected hardware devices is crucial when connecting multiple electronic hardware devices. We started with the idea of constructing an IoT system for subsurface irrigation. We used KiCad version 5.1.2-1 (KiCad is a free software suite, KiCad Services Co., Davis, CA, USA) on a MacBook Pro (3.3 GHz Intel Core i7) to draw our expected schematic and confirm the electrical rules check, as shown in Figure 5. KiCad is a perfect open-source software to create electronic schematic diagrams. KiCad has various stand-alone software tools such as, KiCad project manager, Eeschema (which has been used in this paper), Pcbnew, GrebView, Bitmap2Component, PCB Calculator, and P1 Editor. From our humble perspective, KiCad is mature enough to develop and maintain vast and complex electronic boards. This was behind our motivation to use KiCad for designing our expected schematic design.

Figure 5 represents the detailed schematic KiCad diagram for our system model after running electrical rules check for it. This figure consists of two main parts, the description and the connected electronic devices and sensors. The description part consists of:

- A set of comments related to the connected devices and sensors:
  ○ Comment 1: The Vpulse generator is used as an anemometer to measure air velocity.
  ○ Comment 2: The Vpulse generator is used as a flow meter to measure the water amount per minute.
  ○ Comment 3: The solar radiation is measured by using two separate solar cells.
  ○ Comment 4: The Volumetric Water Content (VWC) is measured by using three VH400 sensors. The average reading of the three sensors is considered.
- ESP8266 NodeMCU board: In our system, we used one ESP8266 NodeMCU board. It is an open-source firmware and development board. We considered using ESP8266 module for its efficacy and simplicity for monitoring and controlling things anywhere in the world. It has 128 KB RAM and 4 MB of Flash memory for data and program storage. The ESP8266 NodeMCU is equipped with 30 pins for interfacing it with the outside world. We used 21 pins to connect all the considered electronic devices and sensors with the ESP8266 NodeMCU board. The used pins are eight power pins (3.3V and GND), 12 multiplexed GPIO pins, and one analog pin. Thus, we still have ten unused pins to do further scaling and improvements for our system in the future using the same board.
- VH400 Volumetric Water Content (VWC) Sensor: We used three VH400 VWC sensors. They are connected to A0, GPIO9, and GPIO10. They are connected to A0 through 74LS151 monolithic data multiplexer. VH400 is used primarily to stop over-watering caused by the traditional watering systems. This sensor introduces precise readings and effective soil moisture content monitoring. Once VH400 is inserted into the soil, it can accurately read in approximately 400 ms (rapid response time). Thus, it can be installed at different depths from the surface. Moreover, it consumes less than 13 mA (low power consumption rate). It is suitable for long-term use. It can be easily interfaced with any system. It can operate from −40 °C to 85 °C.
- DHT11 Digital Temperature & Humidity Sensor: We used two DHT11 sensors. They are connected to GPIO2 and GPIO12. The DHT11 sensor considers the exclusive digital signal acquisition method for sensing humidity and temperature. It is equipped with a high-performance 8-bit microcontroller. It supports long-term stability and high reliability. The accuracy of the DHT11 sensor is ±5% RH and ±2 °C while operating between 20% RH to 90% RH and 0 °C to 50 °C. The features mentioned above are behind our motivation to use such an accurate and efficient sensor for measuring relative humidity and temperature in our water conservation system.
- Flow Meter: We used a flow meter sensor to measure the water flow. It is connected to GPIO4. To calculate the flow rate in L min^−1^, we used a pulse counter to count the number of pulses of the flow meter sensor in exactly one-second intervals. We calculated the number of milliseconds that have passed since the last execution. Then we used the measured number of milliseconds to scale the output. Also, we applied the calibration factor to scale the outcome based on the number of pulses per second per unit of measure (L min^−1^) coming from the sensor. The flow rate is calculated using:
  (1) $$F_{r}=F_{c}\times P_{n}$$
  where *F_r_* is the flow rate (m^3^ h^−1^), *F_c_* is the calibration factor, *P_n_* is pulse number per min.
- Anemometer: This device measures the wind speed and direction. We used a three cups anemometer. It is an aluminum alloyed 4–20 mA current output wind speed sensor. It gives a pulse signal output. The start-up wind speed is 4 to 8 km h^−1^. It is connected to the ESP8266 through GPIO14.
- Solar Cells: We used two single solar cells to measure the solar radiation in the field of the experiment. They are connected to the ESP8266 module through GPIO0 and GPIO13.
- 74LS151 Analog Multiplexer: We used one monolithic data multiplexer. It contains full on-chip binary decoding to select the desired data source. It can choose one-of-eight data sources. It has a strobe input which must be at a low logic level to enable these devices.
- G5Q-1A Electrical Relay: We used two G5Q-1A electrical relays. They are connected to GPIO16. This kind of relay is a single-pole relay. It can switch performance for different loads ideally. The first relay in our model is used to connect the AC motor. The second relay is used to connect the solenoid valve.
- RC1602A-GHW-ESX LCD: We used one LCD in our system. It is mainly used to display some information related to doing an action at the current time. It is connected to the ESP8266 module through GPIO1, GPIO3, and GPIO5.

(2) Software layout

**Figure 6:**
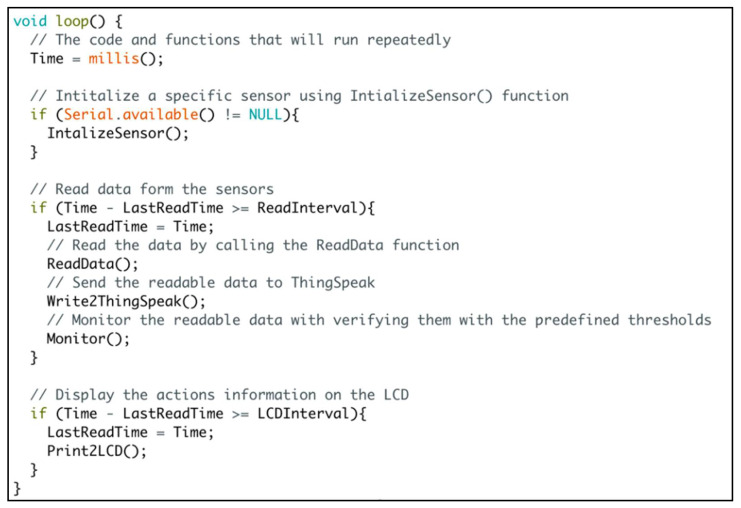
The repeatedly executed code on the ESP8266 NodeMCU board.

We applied the system life cycle to build a successful software by accomplishing the essential requirements for software engineering:

- Requirements: We explicitly defined the measures required for the subsurface irrigation system (VWC, soil temperature, RH, and temperature). Also, the expected output from our software is identified.
- Analysis: The main code is running on the ESP8266 module is divided into multiple functions; each function is responsible for a specific task. As shown in Figure 6, we mainly have five functions:
  ○ *IntializeSensor( )*
  ○ *ReadData( )*
  ○ *Write2ThingSpeak( )*
  ○ *SerialMonitor( )*
  ○ *Print2LCD( )*

- Design: The data variables/objects and operations have been identified.
- Refinement and Coding: the required algorithms and data variables/objects have been implemented.
- Verification: We introduced the verification for our software by comparing the actions taken by our system and another system for collecting the measures manually.
- The detailed description for our functions is as follows:
- *IntializeSensor( )*: It touches the sensors considered in our designed system for resets, calibrations, and manual readings. It should be mentioned that each sensor has a unique name that passed as an argument to the *IntializeSensor( )* function to execute some tasks on that sensor. Also, a getInstruction( ) function is started to collect the user’s instructions for a specific sensor for adjustment purposes.
- *ReadData( )*: once the reading time is reached, the *ReadData( )* function collects the requested measures from all the sensors in our designed system. This is based on the internal library of each sensor.
- *Write2ThingSpeak( )*: it is used to write multiple fields simultaneously to our private channel on the ThingSpeak cloud platform. The designed system does real-time measures based on the small-time interval considered to read the data from the sensors. It should be mentioned that writing the actions to ThingSpeak is done every 10 min. Thus, the average for the measured data during the last 10 min will be written to its designated field to our private channel on ThingSpeak.
- *SerialMonitor( )*: It sends the real measured values from each sensor to the serial monitor at the occurrence of each read interval.
- *Print2LCD( )*: It is responsible for sending the commands that are currently running to the LCD.

### 2.3. Sensors Calibration

The volumetric water content sensor calibration was conducted directly using the soil with different water content in the actual study area. The sensor probe was wholly entered vertically into the soil; then, the readings were recorded. After the sensor was read, the soil sample around the sensor with a diameter of 20 cm and a depth of 20 cm was carefully transported to the laboratory to estimate the actual volumetric water content of the soil samples. The actual volumetric water content was determined based on the gravimetric method by multiplying the gravimetric water content by the soil bulk density, divided by the water density. The amount of water content was determined using a drying oven (LVO-2041P vacuum-drying oven, Dai Han Scientific Co., Ltd., Inchon, Korea) at 105 °C for 48 h. The equations for gravimetric water content and volumetric water content are:

(2) $$\theta_{g}=\frac{S_{w}-S_{d}}{S_{d}}\times 100$$

(3) $$\theta_{V}=\theta_{g}\times \frac{BD_{s}}{D_{w}}$$

where θ_g_ is the gravimetric water content (%), S_w_ is the mass of the wet soil sample (g), S_d_ is the mass of the dry soil sample (g), θ*_v_* is the volumetric water content (%), BD_s_ is soil bulk density (g cm^−3^), and D_w_ is water density (g cm^−3^).

The sensor was calibrated by plotting the sensor readings versus the actual volumetric water content. As the manufacturer calibrated the sensors of temperature & relative humidity, wind speed, and solar radiation energy, the validation of these sensors was conducted by comparing the measured data by the sensors with the observed data by high-quality devices at the same time. After verifying the accuracy of all sensors used, the sensors were applied in the field.

### 2.4. Cloud Layout

**Figure 7:**
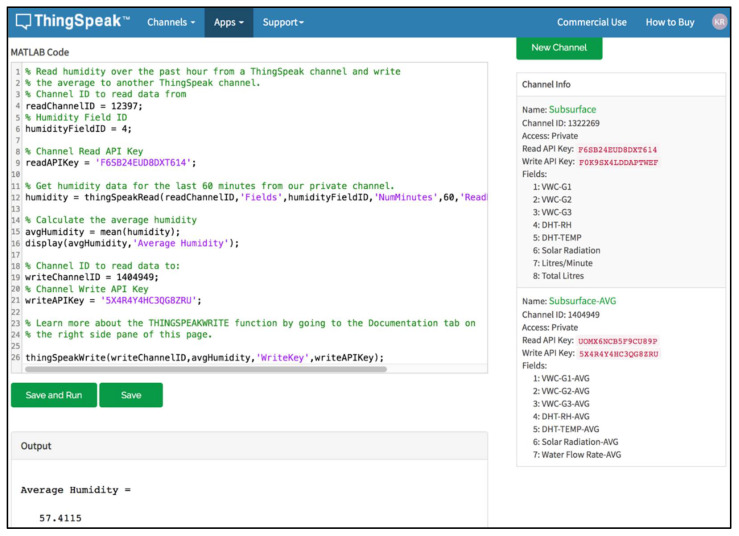
An example for calculating the average humidity over the past hour from our ThingSpeak channel and storing the results in another private channel on ThingSpeak using MATLAB Analysis.

The data is written on our private channel on the ThingSpeak cloud platform using *Write2ThingSpeak( )* function. Then, the cloud platform has three main tasks:

- MATLAB Analysis: It is used to investigate the collected data in our private channel. We employed the MATAB Analysis to calculate and display the average VWC (R1), VWC (R2), VWC (R3), RH, air temperature, solar radiation, and water flow rate over the last 60 min. An example for the average humidity over the past hour is shown in Figure 7. These average results are written to a new private channel and displayed to the user for monitoring. Also, we used it to analyze daily the VWC for each replicate (R1, R2, and R3) and send an email notification to a designated user. It should be mentioned that for simplicity reasons, we removed the screenshots containing the MATLAB Analysis code for the other parameters. If the last value of VWC for a specific replicate is smaller than or equal to the PWP was true six times for one hour, the notification email will be sent to the user. This indicates that there may be a problem in the water pump or any other equipment.
- React: This App is used to perform some actions when the channel data meets a certain condition. We used React App to start the MATLAB Analysis when the last value of VWC for R1, R2, or R3 is less than or equal to PWP (7%). Where the test frequency is every 10 min, and the action is running MATLAB Analysis.
- TalckBack: This App mainly enables the device to act on queued commands. We used to send the commands from our private channel after doing MATLAB Analysis to the ESP8266. Then the ESP8266, by its role, will send the commands to the designated device. For example, when the last VWC value for a specific replicate becomes less than or equal to 15% (Min Setpoint). We created a command that will be sent to the water pump and the solenoid valve to turn them ON. On the other hand, if the last VWC value for a specific replicate becomes greater than or equal to 30% (Max Setpoint), we created a command that will be sent to the water pump and the solenoid valve to turn them OFF.

### 2.5. Experimental Layout

After we designed and implemented the CSIS to keep the soil moisture content next to the field capacity using the optimum amount of the required irrigation water. An experiment was conducted to evaluate the impact of the designed CSIS on the yield of the date palm and water productivity. Because sensor-based scheduling (S-BIS) and time-based scheduling (T-BIS) are considered essential tools that greatly facilitate irrigation scheduling decisions for irrigation water conservation, these methods can be customized based on the plant nature and field characteristics. Therefore, the experiment was conducted on full-grown date palm trees (*Phoenix dactylifera* L.) cv. Khalas at 12 years of age using the S-BIS and T-BIS.

The experimental area had a date palm tree density of 200 palms ha^−1^. The distance of palm-to-palm and row-to-row was 7 m. The S-BIS and T-BIS for the designed SIS were compared with the traditional surface irrigation method applied in the study area for irrigation of date palm trees (Control). In this study, 27 date palm trees were used of approximately similar sizes. The studied date palm trees were divided into 9 similar groups, each group consisting of three trees. Three groups were randomly selected based on a randomized complete block design (RCBD) for each studied subsurface irrigation method of S-BIS and T-BIS and the traditional surface irrigation method.

The irrigation amount was controlled in the S-BIS method based on the VWC in the soil using our cloud platform and IoT system. Because the ON and OFF operations hysteresis of the relays and conductors is a big problem, if the control action (ON/OFF) is based on a threshold, this will frequently change the relays and conductors state ON/OFF. Frequently changing the state negatively affects the life span of the relays, conductors, and other electronic devices. Thus, we employed the Min VWC Setpoint (15%) and the Max VWC Setpoint (30%) to construct a hysteresis band between the ON and OFF. In addition, to keep the soil moisture next to the field capacity for saving irrigation water and preserving the date palm tree from the drought stress of deficit irrigation water.

The irrigation amount was determined in the T-BIS method based on calculated crop evapotranspiration (ETc) and target soil area for the irrigated date palm trees. We applied 50 % of ETc according to the recommendation of Mohammed et al. [1]. The following equation was used for calculating the irrigation water requirement:

(4) $$IWR=\frac{0.5\times ET_{c}\times A_{s}}{1000}$$

where *IWR* is the irrigation water requirement (m^3^ palm^−1^), *ET_C_* is the crop evapotranspiration (mm day^−1^), *As* is the target soil area of a date palm tree.

The crop evapotranspiration (*ET_C_*) was determined using the following formula [8,20]:

(5) $$ET_{c}=K_{c}\times ET_{o}$$

where *ET_C_* is the crop evapotranspiration (mm day^−1^), K_c_ is the crop factor (The mean annual values of the K_c_ were 0.90), and *ET_o_* is the reference evapotranspiration (mm day^−1^).

The reference evapotranspiration ETo was estimated daily based on the FAO Penman-Monteith method [20]. This method required the net radiation at the crop surface, air temperature and relative humidity, wind speed, and other psychrometric constants. These required parameters were determined from the real-time collected data of the experimental site weather by our designed IoT system. The following equation is expressing the method of Penman-Monteith:

(6) $$ET_{o}=\frac{0.408\;\Delta \;\left(R-G\right)+\gamma [900\;u/\left(T+273\right)]\left(e_{s}-e_{a}\right)\;}{\Delta +\gamma \;\left(1+0.34\;u\right)}$$

where *ET_O_* is the reference evapotranspiration (mm day^−1^), ∆ is the slope vapor pressure curve (kPa °C^−1^), R is the net radiation (MJ m^−2^ day^−1^), G is the density of soil heat flux (MJ m^−2^ day^−1^), γ is the psychrometric constant (kPa °C^−1^), u is the wind speed (m s^−1^) at the height of 2 m, T is the air temperature (°C), e_s_ is the pressure of saturation vapor (kPa), and e_a_ is the actual vapor pressure (kPa). The air-water vapor pressure (kPa) was estimated based on hourly and daily measured air temperatures and relative humidity of the study area.

In the traditional surface irrigation methods, four adjustable bubblers are applied to deliver sufficient irrigation water around the date palm trees. The irrigation water is scheduled using an irrigation timer every two days in all year months.

The target soil area was equal to 95% of the mean shaded area of the studied date palm trees. The shaded area was estimated based on the light intercepted fraction by the canopy [9]. The applied shaded area in the experiment was 22 m^2^. The calendar set the irrigation timing under the T-BIS method was conducted according to Mohammed et al. [1] every three days from 1-January to 31 March, every two days from 1 April to 30 September, and every three days from 1-October to 31 December using a programmable timer (model: TM919, Shenzhen HHT Electronics Technology Co., Ltd., Shenzhen, China).

The designed IoT system mounted the actual amount of irrigation water and the cumulative irrigation water throughout the year for the two methods of time-based scheduling (T-BIS) and sensor-based scheduling (S-BIS). The cumulative irrigation water throughout the year was monitored by the readings of the digital flow meter (Model: K24-S, SUNNY, Shandong, China) for the traditional surface irrigation method.

Fertilization was in the exact amounts applied in the field before the experiment, without any change. The fertilization schedule was included nitrogen (3.5 kg tree^−1^), phosphorus (1.5 kg tree^−1^), and potassium (2.5 kg tree^−1^) was used for each date palm tree. These amounts were distributed into similar doses in the irrigation water of the date palm trees (five times per year).

### 2.6. Water Productivity

The water productivity (*WP*) was estimated based on the date palm yield and the cumulative amount of irrigation water using the following equation:

(7) $$WP=\frac{Y}{Wu}$$

where *WP* is water productivity (kg m^−3^), Y is the total marketable yield of date palm (kg), and *Wu* is the annual cumulative amount of irrigation water (m^3^).

### 2.7. Statistical Analysis

Statistical measures were applied to validate the sensors’ accuracy by comparing the measured values taken from the sensor with the observed value simultaneously using standard metrics. These metrics included the determination coefficient, index of agreement, root mean square error, and mean bias error were used for evaluating all applied sensors. The determination coefficient (R-squared correlation (R^2^); Equation (8)) expressed the strength of the measured data fit with the observed data. The index of agreement (d; Equation (9)) was used to measure the degree of the measurement error, which varies between 0 and 1. The d value of 0 indicates no agreement, and 1 indicates a perfect match between the measured and observed data. Root mean square error (RMSE; Equation (10)) was used to compare the difference between values measured by the calibrated sensor and the observed values for each studied parameter. The mean bias error (MBE; Equation (11)) was used to measure the calibration error. The high errors in the measurements produce a low value of MBE. The positive or negative MBE value described the systematic error of the measured values under or over the observed values. Based on the value of MBE, it can be determined whether corrective measures must be taken to correct the calibration bias:

(8) $$r=\frac{n\;(\sum \left(X_{o}\;Y_{m}\right)-\left(\sum X_{o}\right)(\sum Y_{m})}{\sqrt{\left(n\sum X_{o}{}^{2}-{\left(\sum X_{o}\right)}^{2}\right)\left(n\sum Y_{m}{}^{2}-{\left(\sum Y_{m}\right)}^{2}\right)}}$$

where r is Pearson correlation, *n* is the number of the given dataset *X_o_* is the observed data, *Y_m_* is the measured data:

(9) $$\begin{array}{c} d=1-\frac{\sum_{i=1}^{n}{\left(X_{o,i}-Y_{m,i}\right)}^{2}}{\sum_{i=1}^{n}{\left(\left|Y_{o,i}-{\bar{X}}_{o}\right|+\left|X_{o,i}-{\bar{X}}_{o}\right|\right)}^{2}}\;\\ 0\leq d\leq 1 \end{array}$$

where d is the index of agreement, *X_o_* is the observed value, *Y_m_* is the measured value, ${\bar{X}}_{o}$ is the mean observed data:

(10) $$\mathrm{RMSE}=\sqrt{\frac{\sum_{i=1}^{n}{\left(X_{o,i}-Y_{m,i}\right)}^{2}}{n}}$$

where RMSE is the root mean square error, *X_o,_* is the observed value and *Y_m_* is the measured value, *n* is the numbers of values:

(11) $$\mathrm{MBE}=\frac{1}{n}\overset{n}{\underset{i=1}{\sum }}\left(X_{o,i}-Y_{m,i}\right)$$

where MBE is the mean bias error, *X_o_* is the observed value, *Y_m_* is the measured value, and *n* is the number of values.

Data analysis of the date palm yield, water applied, and water productivity was conducted by ANOVA (analysis of variance) using the statistical program of IBM SPSS (SPSS Statistics 24, SPSS Inc., Chicago, IL, USA) at a 0.05 significance level. Tukey test was applied to determine the least significant difference between the experimental means at *p* < 0.05 probability.

## 3. Results and Discussion

### 3.1. Sensors Validation

**Figure 8:**
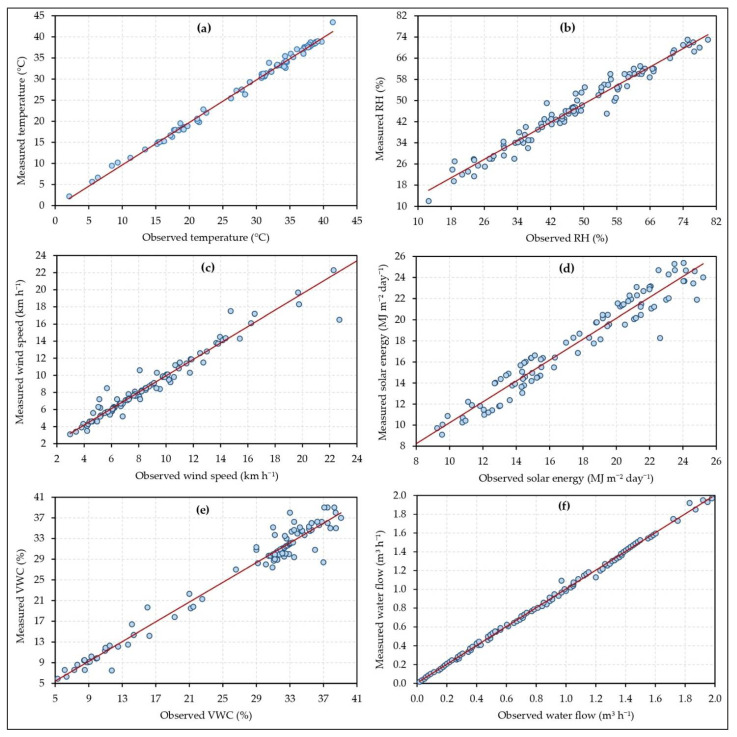
The sensors’ calibration curves of temperature (**a**), relative humidity (**b**), wind speed (**c**), solar energy (**d**), volumetric water content (**e**), and water flow rate (**f**).

The used sensors can lose their precision because of some factors; higher temperatures, high moisture or humidity conditions, subjected to degradation, subjected to external shocks, etc. This loss of precision can be noticed as errors in the measurement process. To tackle the errors in the measurements and apply the needed modification to the sensors or equipment, we applied sensors’ calibration for all used sensors as shown in Figure 8. The figure shows the sensors’ calibration curves for six measures considered in our experiments. The observed (known) values are plotted on the x-axis and the measured values are. plotted on the y-axis for each calibration curve shown in Figure 8. The details of each calibration curve are as follows:

- Figure 8a: This graph represents the temperature calibration curve. The equation of the linear regression model through the temperature points is *y* = 1.007*x* – 0.434, where 1.007 is the slope and 0.434 is the temperature intercept. We used the temperature’s linear regression equation to adjust the temperature measurements collected using the DHT11 sensor. After fitting the measured temperature, the temperature’s R-squared (*R*^2^) is 0.994. This indicates that the used temperature’s linear regression equation produces non-significant differences between the observed temperature and the measured temperature.
- Figure 8b: This graph represents the RH calibration curve. The equation of the linear regression model through the RH points is *y* = 0.872*x* + 5.099, where 0.872 is the slope and 5.099 is the RH intercept. We used the RH’s linear regression equation to adjust the RH measurements collected using the DHT11 sensor. After fitting the measured RH, the RH’s *R*^2^ = 0.963. This indicates that the used RH’s linear regression equation produces non-significant differences between the observed RH and the measured RH.
- Figure 8c: This graph represents the wind speed calibration curve. The equation of the linear regression model through the wind speed points is *y* = 0.96*x* + 0.347, where 0.96 is the slope and 0.347 is the wind speed intercept. We used the wind speed’s linear regression equation to adjust the wind speed measurements collected using the anemometer. After fitting the measured wind speed, the wind speed’s *R*^2^ = 0.983. This indicates that the used wind speed’s linear regression equation produces non-significant differences between the observed wind speed and the measured wind speed.
- Figure 8d: This graph represents the solar energy calibration curve. The equation of the linear regression model through the solar energy points is *y* = 0.991*x* + 0.317, where 0.991 is the slope and 0.317 is the solar energy intercept. We used the solar energy’s linear regression equation to adjust the solar energy measurements collected using the solar cells. After fitting the measured solar energy, the solar energy’s *R*^2^ = 0.944. This indicates that the used solar energy’s linear regression equation produces non-significant differences between the observed solar energy and the measured solar energy.
- Figure 8e: This graph represents the VWC calibration curve. The equation of the linear regression model through the VWC points is *y* = 0.872*x* + 5.0949, where 0.872 is the slope and 5.0949 is the VWC intercept. We used the VWC’s linear regression equation to adjust the VWC measurements collected using the VH400 sensor. After fitting the measured VWC, the VWC’s *R*^2^ = 0.963. This indicates that the used VWC’s linear regression equation produces non-significant differences between the observed VWC and the measured VWC.
- Figure 8f: This graph represents the water flow calibration curve. The equation of the linear regression model through the water flow points is *y* = 0.999*x* + 0.004, where 0.999 is the slope and 0.004 is the water flow intercept. We used the water flow’s linear regression equation to adjust the water flow measurements collected using the flow meter. After fitting the measured water flow, the water flow’s *R*^2^ = 0.998. This indicates that the used water flow’s linear regression equation produces non-significant differences between the observed water flow and the measured water flow.

Table 2 shows the comparison between the observed values and the measured values by the temperature & relative humidity sensor, solar energy sensor, wind speed sensor, volumetric water content sensor, and water flow sensor using the standard statistical metrics of the index of agreement (d), root mean square error (RMSE), and mean bias error (MBE). From this table, it was noted that the calibration by the manufacturer of the used temperature & relative humidity sensor, solar energy sensor, wind speed sensor, and water flow sensor was achieved the required accuracy within the tested parameters. The calibration that we carried out on the volumetric water content sensor also achieved the required accuracy for measuring water content in the field.

### 3.2. Cloud Monitoring

**Figure 9:**
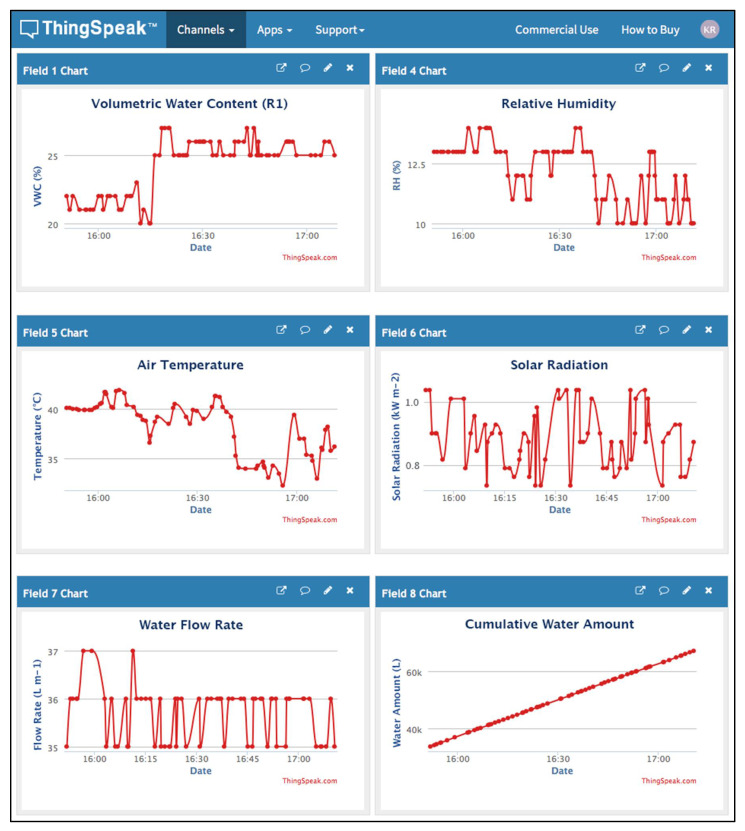
Some fields from our private channel on the ThingSpeak cloud platform. It represents users’ monitoring interface.

The ESP8266 module uploads the collected measurements every minute using *Write2ThingSpeak( )* function to our private channel on the ThingSpeak cloud platform, as shown in Figure 9. Our private channel consists of eight fields. We selected only six fields to be shown here for simplicity reasons. The detailed description of each field in Figure 9 is as follows:

- Field 1 Chart: It shows the average real-time VWC percentage collected from three VH400 sensors for replicate R1. Each of these sensors is installed at 0.3 m depth between two subsurface irrigation units. The minimum VWC setpoint (Min VWC Setpoint) is 15%, and the maximum VWC setpoint (Max VWC Setpoint) is 35%. Our CSIS start sending instructions to the water pump to work and feed the replicate with water when the VWC average value is smaller than or equal to 15%. During the feeding time, the average VWC is also monitored. When the VWC average value becomes greater than or equal 30%, the CSIS will send instructions to the water pump to turn off. As shown in this chart, the VWC is ranging between 20% to 27% within two hours. We avoided the hysteresis ON and OFF for our devices by letting the ON and OFF instructions run between the Min Setpoint and Max Setpoint.
- Field 2 Chart: Shows the average real-time VWC percentage collected from three VH400 sensors for replicate R2.
- Field 3 Chart: Shows the average real-time VWC percentage collected from three VH400 sensors for replicate R3 (not shown in Figure 9 for simplicity reasons).
- Field 4 Chart: Shows the average real-time RH percentage collected from two DHT11 sensors for the study area. This field is used for monitoring purposes. As shown in this chart, the RH is ranging between 10% to 14% within two hours.
- Field 5 Chart: Shows the average real-time air temperature collected from two DHT11 sensors for the study area. This field is used for monitoring purposes. As shown in this chart, the air temperature is ranging between 36 °C to 45 °C within two hours.
- Field 6 Chart: Shows the average solar radiation collected from two solar cells in the study area. This field is used for monitoring purposes. As shown in this chart, the solar radiation is ranging between 10.685 MJ m^−2^ to 12.192 MJ m^−2^ within two hours.
- Field 7 Chart: Shows the water flow rate per minute collected from the flow meter for each replicate. This field is used for monitoring purposes to make sure that the AC motor is working well. As shown in this chart, the flow rate is ranging between 35 L/m to 37 L/m within two hours. It should be mentioned that the average flow rate is always 36 L/m.
- Field 8 Chart: It shows the cumulative water amount applied for each replicate. This field is used for monitoring purposes to make sure that each replicates properly received the water amount. As shown in this chart, the cumulative water amount ranges from 20,638 L to 70,914 L within two hours.

### 3.3. Meteorological Data of the Study Area

**Figure 10:**
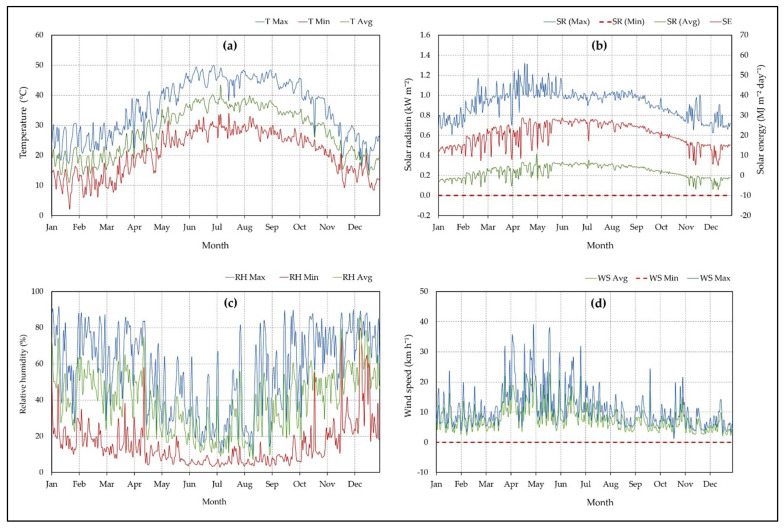
Daily average (Avg), minimum (Min), and maximum (Max) of the temperature (**a**), solar radiation (**b**), relative humidity (**c**), and wind speed (**d**) over the year 2020 in the study area.

The required meteorological data for estimating the reference evapotranspiration were real-time tracked and recorded using our cloud-based IoT system every day over the experimental year of 2020. Figure 10 shows the daily average, minimum, and maximum of the temperature (°C) as shown in Figure 10a, solar radiation (MJ m^−2^ day^−1^) as shown in Figure 10b, percentage of relative humidity as shown in Figure 10c, and wind speed in m h^−1^ as shown in Figure 10d over the experimental season of 2020 of the study area. Since the study area is very arid and where rain is scarce, the rainfall was not considered.

The data showed that the highest monthly mean temperature was 37.56 °C, 37.55 °C, and 37.3 °C during the summer months of June, August, and July, respectively, while the lowest monthly mean temperature was 17.85 °C, 18.4 °C, and 18.55 °C during the winter months of February, December, and January, respectively, as shown in Figure 10a. The highest monthly mean solar radiation energy was 25.1 MJ m^−2^ day^−1^ in May, while the lowest monthly mean solar radiation energy was 14.1 MJ m^−2^ day^−1^ in December, as shown in Figure 10b. The highest monthly mean relative humidity was 63.5% and 60.2% during December and February, respectively. The lowest monthly mean relative humidity was 23.2 and 27.3% during June and July, respectively, as shown in Figure 10c. The highest monthly mean wind speed was 9.33 km h^−1^ and 8.5 km h^−1^ during July and June, respectively, while the lowest monthly mean wind speed was 6.08 km h^−1^ and 6.42 km h^−1^ during October and December, respectively, as shown in Figure 10d.

### 3.4. Sensor-Based Irrigation Schedule

#### 3.4.1. Timing of Actuators Operations

The appropriate timing was established for each operation to ensure the optimum operation of the actuators of the S-BIS method. We used the Min VWC Setpoint (15%) and the Max VWC Setpoint (30%) to construct a hysteresis band between the ON and OFF. Based on these setpoints, the solenoid valve will On or Off.

**Figure 11:**
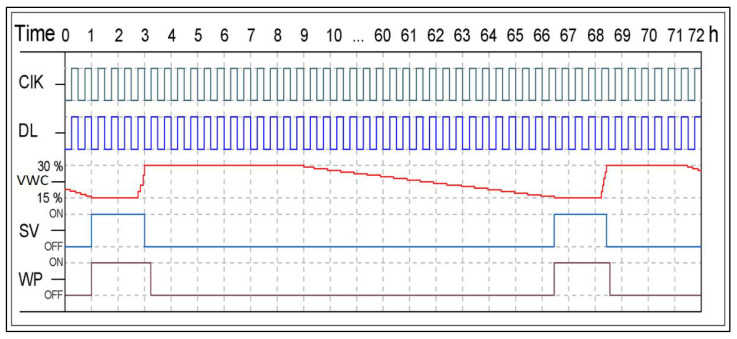
The ON and OFF control action timing diagram. ClK is clock of timing, DL is data logging, VWC is the measured volumetric water content, SV is the solenoid valve, and WP is the water pump.

Figure 11 shows the time interval of three days in hours from 0 to 72 h under the S-BIS method. The clock of timing (ClK) is shown in green color. The data logging (DL) is shown in blue color. The actual measured VWC is shown in red color. The figure shows that the solenoid valve (SV) operations (ON/OFF) timing is light blue. The water pump (WP) operations (ON/OFF) timing is shown in brown color. We considered that the VWC was about 18% at time 0. VWC was continuously decaying over time while the SV and WP are turned OFF. When the VWC becomes smaller than or equal to the Min VWC Setpoint (15%) at time 1, the SV and WP will be turned ON. They will work for two hours. During the ON time of the SV and WP, the VWC is increasing. When the VWC becomes greater than or equal to the Max VWC Setpoint (30%), the SV will be turned OFF, while the WP is still turned ON for 10 min after turning the SV OFF to compensate for the water pressure. Then, the VWC will be stable at 30% or maybe a little higher for about six hours (until time 9). After that, the VWC will start to decay over time as the SV and WP are turned OFF. The VWC will continue decaying until reaching the Min VWC Setpoint (15%) at time 66.5, the SV and WP will be turned ON, and the previously mentioned operations will be automatically repeated.

#### 3.4.2. Monitoring of the Volumetric Water Content

**Figure 12:**
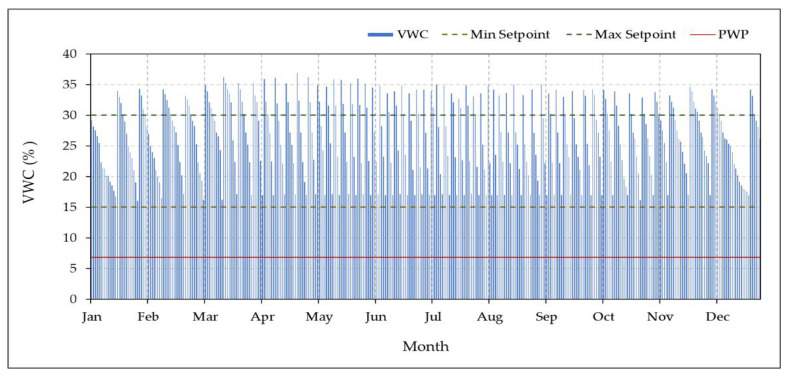
The measured volumetric water content of the controlled subsurface irrigation system soil with sensor-based irrigation method of over the year 2020 in the experimental site. The VWC was controlled between the Min Setpoint (15%) and the Max Setpoint (30%). PWP is the permanent welting point of the soil in the experimental site.

We tracked, controlled, and recorded the VWC for one date palm tree from each group (each group has three date palm trees) over a year using the designed CSIS with the S-BIS method, as shown in Figure 12. The figure shows the average daily value of three VH400 sensors installed around the roots of the date palm tree. The months are represented horizontally over the x-axis, and the daily VWC is represented vertically over the y-axis. The considered Min Setpoint of VWC is 15%. If the measured VWC becomes smaller than or equal to the Min Setpoint, CSIS will turn the water pump and the solenoid valve ON to add water for three date palm trees. Also, the considered Max Setpoint of VWC is 30%. If the measured VWC becomes greater than or equal to the Max Setpoint, CSIS will turn the water pump and the solenoid valve OFF. It should be mentioned that the permanent wetting point (PWP) in our experimental site is 6.8%, where PWP is the minimum VWC in the soil that the date palm tree requires not to wilt. The VWC is increased above the max setpoint 35% after turning the water pump and the solenoid valve OFF for a short time due to water infiltration from the saturated soil around the subsurface irrigation units.

#### 3.4.3. Monitoring of Irrigation Water Applied

**Figure 13:**
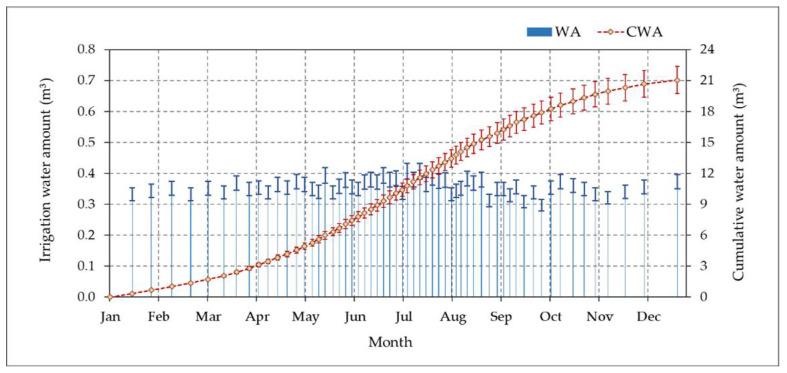
The mean applied irrigation water amount (WA) and the mean cumulative applied water amount in cubic meter (CWA) over the year 2020 in the experimental site using the designed controlled subsurface irrigation system (CSIS) with the sensor-based irrigation scheduling method (S-BIS).

We tracked and recorded the water amount used for studied date palm trees and the cumulative water amount used over a year under the designed CSIS with the S-BIS method. The mean water amount and the mean cumulative water used are shown in Figure 13. The months are represented horizon-tally over the x-axis, and the daily water amount and cumulative water amount are represented vertically over the y-axis. The mean cumulative water amount over a year was 21.04 m^3^. In the S-BIS method, it was observed that the irrigation periods were not fixed during the study period; they converged or diverged according to the VWC in the soil, as shown in Figure 13. In the summer months, irrigation periods were closed due to the extreme weather conditions in these months at the experimental site, in which evapotranspiration was high.

### 3.5. Time-Based Irrigation Schedule

#### 3.5.1. Crop Evapotranspiration

**Figure 14:**
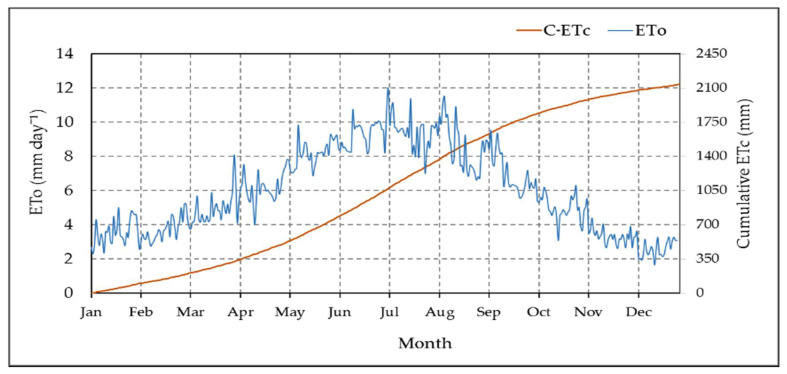
The calculated reference evapotranspiration (ETo) and the cumulative crop evapotranspiration (C-ETc) over the year 2020 in the experimental site.

The irrigation amount was determined in the T-BIS method based on the ETc. In this study, the ETc was estimated based on crop factor (0.95) and the calculated ETo [8,9,19]. We used the Penman-Monteith equation (Equation (6)) and our collected meteorological data of the study area using the designed cloud IoT system to estimate the ETo. The mean values of the daily ETo rates in our experimental site peaked in the summer months from May to August, as shown in Figure 14. The results presented in this figure showed that the highest mean value of ETo was 9.43 mm day^−1^, 9.19 mm day^−1^, and 8.69 mm day^−1^ during the summer months of July, June, and August, respectively, while the lowest mean values of ETo was 2.73 mm day^−1^, 3.52 mm day^−1^, and 3.57 mm day^−1^ during the winter months of December, January, and February, respectively. We calculated the daily ETc based on Equation No, then the cumulative annual value of ETc was calculated. The annual cumulative value of ETc was 2137 mm, as shown in Figure 14. The obtained data of ETo was similar to the resulted data presented by the authors in [1,11,32].

#### 3.5.2. Monitoring of Irrigation Water Applied

**Figure 15:**
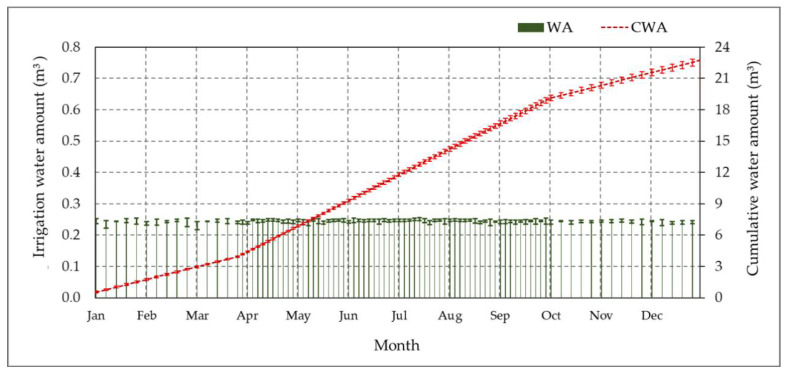
The mean applied water amount (WA) and the mean cumulative supplied water amount (CWA) in cubic meters over the year 2020 in the experimental site using the designed controlled subsurface irrigation system (CSIS) with the time-based irrigation scheduling method (T-BIS).

The amount of applied irrigation in the T-BIS throughout the study period was estimated as a percentage of the ETc (50% of ETc) based on a calendar set for irrigation timing. Therefore, unlike the S-BIS method, the time interval was fixed based on each month. We tracked and recorded the actual water amount for studied date palm trees and the cumulative water amount used over a year under the designed CSIS with the T-BIS method. The mean water amount and the mean cumulative water used are shown in Figure 15. The months were represented horizontally over the x-axis, and the daily water amount and cumulative water amount were presented vertically over the y-axis. It should be mentioned that the decade is changing in the T-BIS based on fixed time intervals for each month. Therefore, the cumulative water intake line was straight for each period. The mean cumulative water amount used over a year was 22.76 m^3^. It is noticed that the cumulative value in the T-BIS method is higher than the T-BIS method by 1.72 m^3^ palm^−1^. Generally, the annual cumulative irrigation water applied of the date palm tree using the designed CSIS with the S-BIS or T-BIS was below the range reported by the authors in [1,9,12,18,20,27,33].

### 3.6. Date Palm Yield and Water productivity

Table 3 shows the impact of S-BIS and T-BIS methods of the designed CSIS compared with the TSI method (Control) on the date palm yield and the applied water amount and its productivity. There was a significant difference, regarding the actual annual irrigation depth (ANOVA, F2, 26, = 8495, *p* < 0.001), amount of cumulative applied irrigation water (ANOVA, F2, 26, = 8735, *p* < 0.001), the total marketable yield of date palm (ANOVA, F2, 26, = 47.58, *p* < 0.001), water productivity (ANOVA, F2, 26, = 677.2, *p* < 0.001) for S-BIS and T-BIS methods of the designed CSIS and the TSI method. The highest values of marketable yield and water productivity were noted at the CSIS with the S-BIS method. In contrast, the lowest values were recorded at the traditional surface irrigation method. The date palm trees irrigated by the designed CSIS with the S-BIS method showed a significant decrease in the irrigation water applied (44.7% of ETc) compared with the T-BIS method (48.4 % of ETc) and the traditional surface irrigation method (124.8 of ETc), though the total marketable yield was more than that of the S-BIS and the traditional surface irrigation methods. Generally, the S-BIS and T-BIS of CSIS reduced the applied water depth to 44.73% and 48.4% of ETc, respectively, compared with the traditional surface irrigation used 124.8% of ETc. Therefore, the S-BIS and T-BIS of CSIS reduced the applied irrigation water amount to 64.1% and 61.2%, respectively, compared with traditional surface irrigation (TSI).

The high increases in date palm yield and water productivity were due to the high efficiency of the designed CSIS compared to the traditional surface irrigation methods. The designed CSIS efficiently distributed the irrigation water directly in the functional root zone of the date palm tree without runoff of irrigation water. In addition, the CSIS system does not only stops the runoff of irrigation water but also prevents any water loss that occurs through water evaporation from the soil surface. The CSIS with S-BIS was kept the moisture of the soil next to the field capacity with saving irrigation water compared to T-BIS. The increases in date palm yield and water productivity with low irrigation water consumption could be due to the optimal availability of water in the S-BIS method that enhanced balanced root growth and improved soil nutrient uptake, as mentioned in [34,35]. The authors in [36] found that a subsurface drip irrigation system was the most useful regarding date palm yield and water productivity. The impact of irrigation water availability on plant growth is due to the differences in carbon uptake, stomatal conductance, and turgor pressure of plant tissues. Therefore, the controlled application of water improves fruit yield and quality, which varied at growth stages of vegetative and productive, and severity and duration of deficit water [37]. Authors in [1] mentioned that the subsurface irrigation system is characterized by water application at low intensity directly underneath the soil surface in the functional root zone of the date palm tree.

Modern automatic irrigation sensors and devices are essential for the development of traditional irrigation systems for water conservation. The irrigation systems embedded with modern water-saving irrigation systems have been further developed to improve water productivity [4,17,38,39]. Our study added a solution for addressing and reducing the disadvantages of water-saving subsurface irrigation methods using IoT applications for overcoming difficulty controlling system operation. In addition, the water applications utilizing the subsoil irrigation systems are largely unseen for monitoring, managing, and evaluating irrigation events. The irrigation scheduling in our study was based on the volumetric water content sensors in the CSIS with S-BIS for overcoming the possibility that emitter flow rates can exceed the ability of the soil to distribute the irrigation water in the functional root zone. Compared with the constant irrigation depth, which can be designed and estimated by computer programs or practical experience in previously published investigations [1,40,41], our smart irrigation system showed improvements for water productivity and date palm yield. Although using a constant irrigation depth can ease and simplify irrigation operation, not applying the actual irrigation depth of irrigation water uptake usually results in more or less irrigation [20,42,43]. In addition, there is a difficulty in controlling the irrigation water using the traditional subsurface irrigation systems, where the estimation of water was based on evapotranspiration calculated from previous years, not from real-time weather results. This necessitated the use of the IoT to monitor important weather parameters such as solar radiation intensity, temperature, relative humidity, and wind speed to real-time evapotranspiration estimation. Therefore, our study ensured good management of the subsurface irrigation system to avoid the deficit irrigation and date palm yield and quality reductions or over-irrigation using CSIS with T-BIS.

## 4. Conclusions

A fully automated controlled subsurface irrigation system (CSIS) for improving the irrigation management of date palms in an arid region was designed, built, and evaluated. The designed CSIS with sensor-based irrigation scheduling (S-BIS) has a high positive impact on the marketable yield of date palm, water consumption, and water productivity, followed by time-based irrigation scheduling (T-BIS). The positive effects of the S-BIS method were due to the high efficiency of the designed CSIS that enhanced root growth and improved the soil nutrient uptake in the functional root zone compared to the TSI method. Generally, the S-BIS and T-BIS of CSIS reduced the applied irrigation water amount to 64.1% and 61.2%, respectively, compared with traditional surface irrigation (TSI). The water productivity at the CSIS with S-BIS (1.783 kg m^−3^) and T-BIS (1.44 kg m^−3^) methods was significantly higher compared to the TSI (0.531 kg m^−3^). The estimated irrigation water based on our results is 4208 m^3^ ha^−1^ or 4552 m^3^ ha^−1^ using the designed CSIS with S-BIS or T-BIS, respectively, compared to 11742 m^3^ ha^−1^ by using TSI method (200 palm ha^−1^). Moreover, we expect that the production costs of date fruits may be lowered by reducing certain farming practices such as irrigation management and weeding by using the designed CSIS with S-BIS compared to TSI. Finally, we concluded that the designed CSIS could be recommended for irrigation water management of date palms in arid and semi-arid regions for its high efficiency in these conditions. Further investigations are needed to study the impact of the modern CSIS on different types of fruit trees and other soil types.

## Figures and Tables

**Table 1:** Physical and hydraulic properties of the sandy loam soil in the experimental site.

Soil Depth	Particle Size Distribution			BD (g cm^−3^)	Fc (%)	PWP (%)	pH	EC_s_ (dS m^−1^)	HC (cm h^−1^)
	Sand (%)	Silt (%)	Clay (%)						
0–25	65	19	16	1.57	15.6	6.8	8.1	3.22	4.8
25–50	67	18	15	1.55	16.5	6.7	7.8	3.21	4.7
50–75	66	19	15	1.58	16.2	7.1	7.9	3.18	4.9
75–100	69	18	13	1.59	16.7	6.5	8.1	3.24	4.9
Mean	66.8	18.5	14.8	1.6	16.3	6.8	8.0	3.21	4.8
St. Dev.	1.71	0.58	1.26	0.02	0.48	0.25	0.15	0.03	0.10

BD is bulk density, Fc is the field capacity, PWP is the permanent wilting point, pH is the concentration of hydrogen ions, EC_s_ is the electrical conductivity, and HC is the hydraulic conductivity.

**Table 2:** Statistical metrics values resulting from comparing the measured values of the temperature & relative humidity sensor, solar energy sensor, wind speed sensor, volumetric water content sensor, and water flow sensor with the observed values of the required weather, soil, water parameters of temperature (°C), relative humidity (%), wind speed (m h^−1^), Solar energy (MJ m^−2^ day^−1^), soil water content (%), and water flow (m^3^ h^−1^) in the study area during the season of 2020.

Parameters	Statistical Metrics		
	Index of Agreement (d)	Root Mean Square Error (RMSE)	Mean Bias Error (MBE)
Temperature	0.998	0.780	−0.234
Relative humidity	0.986	0.497	−1.026
Wind speed	0.985	1.114	0.163
Solar energy	0.999	0.024	0.004
Soil water content	0.991	2.001	−0.527
Water flow	0.995	0.978	−0.053

The numbers of the tested values (*n*) were 100 values for all studied parameters.

**Table 3:** Annual applied water (mm year^−1^ palm^−1^), amount of applied water (m^3^ year^−1^ palm^−1^), the total yield of the date palm tree (kg palm-1), and water productivity (kg m^−3^) under sensor-based irrigation schedule (S-BIS) and time-based irrigation schedule (T-BIS) methods of the controlled subsurface irrigation system (CSIS) compared with the traditional surface irrigation (TSI as a Control) method of the experimental season of 2020.

Parameters	Irrigation Methods		
	CSIS		TSI (Control)
	S-BIS	T-BIS	
Annual applied water in depth	956.3 ± 52.1 ^A^	1034.6 ± 13.1 ^B^	2668.7 ± 9.5 ^C^
Amount of applied water	21.03 ± 1.13 ^A^	22.76 ± 0.29 ^B^	58.71 ± 0.21 ^C^
Total yield of date palm tree	37.57 ± 0.92 ^A^	34.86 ± 2.03 ^B^	30.97 ± 1.13 ^C^
Water productivity	1.783 ± 0.08 ^A^	1.440 ± 0.09 ^B^	0.531 ± 0.03 ^C^

The least significant difference between the means was determined using the Tukey test. Figures sharing the same letter in a row are non-significant at (*p* ˂ 0.05) probability.

## Data Availability

Not applicable.
